# Effect of Immune Checkpoint Inhibitor Therapy on Biventricular and Biatrial Mechanics in Patients with Advanced Cancer: A Short-Term Follow-Up Study

**DOI:** 10.3390/jcm15020762

**Published:** 2026-01-16

**Authors:** Andrea Sonaglioni, Emanuela Fossile, Nicoletta Tartaglia, Gian Luigi Nicolosi, Michele Lombardo, Massimo Baravelli, Paola Muti, Pier Francesco Ferrucci

**Affiliations:** 1 Division of Cardiology, IRCCS MultiMedica, 20123 Milan, Italy; michele.lombardo@multimedica.it (M.L.); massimo.baravelli@multimedica.it (M.B.); 2 Department of Oncology, IRCCS MultiMedica, 20123 Milan, Italy; emanuela.fossile@multimedica.it (E.F.); pierfrancesco.ferrucci@multimedica.it (P.F.F.); 3 Hospital Pharmacy, IRCCS MultiMedica, 20123 Milan, Italy; nicoletta.tartaglia@multimedica.it; 4 Division of Cardiology, Policlinico San Giorgio, 33170 Pordenone, Italy; gianluigi.nicolosi@gmail.com; 5 Department of Biomedical, Surgical and Dental Sciences, University of Milan, 20122 Milan, Italy; paola.muti@unimi.it; 6 IRCCS MultiMedica, 20138 Milan, Italy

**Keywords:** ICI, cardiac mechanics, subclinical dysfunction, speckle-tracking echocardiography, prognostic risk stratification

## Abstract

**Background**: Immune checkpoint inhibitors (ICIs) improve cancer outcomes but may cause cardiovascular toxicity, including early subclinical myocardial injury. Conventional echocardiography has limited sensitivity, whereas speckle-tracking echocardiography (STE) allows for early detection of myocardial deformation. Data on short-term ICI-related effects on biventricular mechanics are limited, and atrial function remains poorly characterized. This study evaluated the early impact of ICI therapy on biventricular and biatrial mechanics using STE in patients with advanced cancer. **Methods**: In this prospective, single-center study, 28 consecutive patients with advanced cancer undergoing ICI therapy were followed for 3 months. Clinical, laboratory, electrocardiographic, and echocardiographic assessments were performed at baseline, 1 month, and 3 months. STE was used to assess left ventricular global longitudinal strain (LV-GLS) and circumferential strain; right ventricular GLS (RV-GLS); and left and right atrial reservoir, conduit, and contractile strain parameters. Subclinical LV dysfunction was defined as a relative LV-GLS reduction >15%. Logistic and Cox regression analyses identified predictors of strain impairment and adverse clinical events. **Results**: Conventional echocardiographic parameters, including left ventricular ejection fraction, remained stable. In contrast, LV-GLS declined progressively from 20.7 ± 2.1% to 17.6 ± 2.7% at 3 months (*p* = 0.002), with subclinical LV dysfunction observed in 85.7% of patients. RV-GLS also deteriorated despite preserved TAPSE. Both left and right atrial strain and strain-rate parameters showed an early and marked decline, accompanied by increased left atrial stiffness despite unchanged atrial volumes. Older age and higher neutrophil-to-lymphocyte ratio (NLR) were associated with LV-GLS impairment. Over a mean follow-up of 5.4 ± 3 months, baseline LV-GLS independently predicted adverse clinical events and mortality. Optimal cut-off values were 67 years for age, 4 for NLR, and 19.5% for LV-GLS. **Conclusions**: Short-term ICI therapy is associated with early, diffuse subclinical myocardial dysfunction involving both ventricles and atria, detectable only by STE. Comprehensive biventricular and biatrial strain assessment may enhance early cardio-oncology surveillance and risk stratification in ICI-treated patients.

## 1. Introduction

Immune checkpoint inhibitors (ICIs) have become a cornerstone of modern immuno-oncology and have demonstrated significant survival benefits across a broad spectrum of malignancies, both in (neo)adjuvant and metastatic settings [1,2]. Unlike conventional anticancer therapies, ICIs exert their therapeutic effect by restoring and enhancing host immune responses against tumor cells [3]. To date, the U.S. Food and Drug Administration has approved agents targeting three major immune checkpoints—cytotoxic T-lymphocyte-associated antigen 4 (CTLA-4), programmed cell death protein 1 (PD-1) and its ligand PD-L1, and lymphocyte activation gene 3 (LAG-3)—either as monotherapy or in combination with other ICIs, chemotherapy, or targeted therapies.

Despite their clinical efficacy, ICIs are associated with immune-related adverse events (IRAEs), which may involve multiple organ systems, including the cardiovascular system [4,5,6,7]. Among cardiovascular IRAEs, ICI-related myocarditis represents the most severe manifestation and typically occurs within the first three months after treatment initiation. Although relatively uncommon, with a reported prevalence ranging from 0.09% to 2.4%, ICI-related myocarditis carries a high mortality rate of up to 25–50% [8,9,10,11,12,13]. Increased awareness of this potentially fatal complication has stimulated growing interest in identifying less severe or subclinical forms of ICI-related myocardial injury [14].

Conventional transthoracic echocardiography (TTE), with assessment of left ventricular ejection fraction (LVEF), has limited sensitivity for the early detection of myocardial dysfunction, as contractile impairment may already be present despite preserved LVEF values [15,16]. Speckle-tracking echocardiography (STE) has emerged as an advanced imaging technique capable of overcoming these limitations by quantifying myocardial deformation (strain) and strain rate in longitudinal, circumferential, and radial directions during systole and diastole [17,18]. Using STE, myocardial mechanical properties of both ventricles and atria can be accurately assessed. In particular, early impairment of left ventricular global longitudinal strain (LV-GLS), the most widely used STE-derived parameter, has been consistently shown to precede LVEF reduction in a variety of clinical settings [19,20,21]. Moreover, myocardial strain abnormalities affecting both ventricular and atrial chambers have been shown to correlate with the degree of myocardial edema and fibrosis documented by endomyocardial biopsy [22,23].

Over the past few years, several imaging studies have evaluated myocardial strain parameters in patients undergoing ICI therapy at baseline and during short-term follow-up, reporting heterogeneous and sometimes conflicting results [24,25,26,27,28,29,30,31,32]. While the majority of studies observed a significant reduction in strain values during follow-up compared with accepted reference ranges [33], some investigations did not detect significant changes in LV-GLS after ICI exposure [26,32]. Notably, only a limited number of studies have simultaneously assessed left and right ventricular myocardial strain, consistently demonstrating early attenuation of biventricular mechanics following ICI therapy [24,26,29].

In contrast to ventricular deformation analysis, atrial myocardial mechanics have received very limited attention in the context of ICI therapy. Although STE enables a comprehensive evaluation of atrial function, including reservoir, conduit, and contractile phases, atrial strain assessment has been only sporadically incorporated into imaging protocols of ICI-treated patients and generally limited to isolated reports or secondary analyses [26]. Consequently, the prevalence, temporal evolution, and clinical implications of left and right atrial mechanical impairment following ICI exposure remain largely undefined. Given the strong functional interdependence between atrial and ventricular chambers and the high sensitivity of atrial strain to early myocardial stiffness, inflammation, and diastolic dysfunction, the paucity of data on atrial mechanics represents a relevant gap in current cardio-oncology research.

On the basis of these considerations, we hypothesized that ICI therapy may induce early, subclinical myocardial dysfunction involving not only both ventricles but also both atria. Accordingly, the present study aimed to evaluate the short-term effects of ICI therapy on biventricular and biatrial mechanics using advanced strain imaging in patients with advanced cancer.

## 2. Materials and Methods

### 2.1. Study Population

The present observational, prospective, monocentric study was conducted at Ospedale San Giuseppe MultiMedica IRCCS between 5 May 2025 and 1 August 2025 and was designed to evaluate the effect of ICI therapy on biventricular and biatrial mechanics in patients with advanced cancer over a three-month follow-up period.

Inclusion criteria were adult patients (>18 years) with histologically confirmed advanced malignancies of the lung, breast, skin, or kidney, consecutively referred to our Institution and scheduled to initiate ICI therapy, either in the neoadjuvant or adjuvant setting, or for metastatic disease. All patients were recruited from the Division of Oncology of Ospedale San Giuseppe MultiMedica.

Exclusion criteria were age ≤ 18 years; absence of advanced cancer or lack of indication for ICI therapy; hemodynamic instability resulting from atrial tachyarrhythmias associated with arterial hypotension, acute heart failure, acute respiratory failure, or acute renal failure; inadequate echocardiographic acoustic windows, defined as insufficient endocardial border visualization resulting in unreliable ventricular and atrial speckle-tracking analysis; and refusal to provide informed consent for participation in the study.

This study was conducted in accordance with the ethical standards of the Declaration of Helsinki and received approval from the local Ethics Committee (reference no. 211/25). All participants provided written informed consent before participation.

### 2.2. Clinical, Laboratory, and Electrocardiographic Assessment

At baseline evaluation, comprehensive clinical, laboratory, and electrocardiographic data were collected. Oncological characteristics were recorded, including cancer type, disease stage (metastatic or nonmetastatic), and ICI treatment setting (neoadjuvant, adjuvant, or metastatic). Demographic data included age, while anthropometric measurements comprised body surface area (BSA) and body mass index (BMI). Clinical assessment included heart rate and blood pressure measurements.

Laboratory analyses encompassed a complete peripheral blood count, from which hemoglobin concentration, red cell distribution width (RDW), and neutrophil-to-lymphocyte ratio (NLR) were derived for subsequent analyses, as well as serum creatinine with estimated glomerular filtration rate (eGFR) [34], C-reactive protein (CRP), high-sensitivity cardiac troponin T (hs-TnT), and N-terminal pro–brain natriuretic peptide (NT-proBNP). A standard 12-lead electrocardiogram (ECG) was obtained in all participants.

### 2.3. Conventional Echocardiographic Assessment

All transthoracic echocardiographic examinations were performed at baseline and during follow-up on the same day as clinical evaluation by a single experienced cardiologist to minimize inter-observer variability. All echocardiographic studies were performed using a commercially available Philips Sparq ultrasound system (Philips, Andover, MA, USA) equipped with a 2.5 MHz transducer. Image acquisition was conducted with patients in the left lateral decubitus position, and measurements were obtained in accordance with the recommendations of the American Society of Echocardiography and the European Association of Cardiovascular Imaging [35,36].

The following two-dimensional echocardiographic parameters were recorded: aortic root and ascending aorta diameters measured using the leading-edge–to-leading-edge method; relative wall thickness (RWT), calculated as twice the posterior wall thickness divided by left ventricular end-diastolic diameter; left ventricular mass index (LVMi), derived using the Devereux formula; indexed left ventricular end-diastolic and end-systolic volumes (LVEDVi and LVESVi); and LVEF, assessed using the biplane modified Simpson method as an indicator of systolic function [35]. Additional measurements included left atrial volume index (LAVi), right ventricular inflow tract (RVIT) diameter, tricuspid annular plane systolic excursion (TAPSE) as a marker of right ventricular systolic performance, and inferior vena cava (IVC) diameter during spontaneous respiration.

Doppler analysis included transmitral early-to-late diastolic flow velocity ratio (E/A) and the ratio between early mitral inflow velocity and averaged mitral annular early diastolic velocity (E/e′) as indices of left ventricular diastolic function and filling pressures, respectively [36]. Systolic pulmonary artery pressure (sPAP) was estimated using the modified Bernoulli equation (sPAP = 4 × TRV^2^ + estimated right atrial pressure) [37]. The severity of concomitant valvular heart disease was graded according to current AHA/ACC guidelines [38].

### 2.4. Myocardial Deformation Analysis by Speckle-Tracking Echocardiography

Following completion of conventional TTE, myocardial deformation analysis was performed using high-quality two-dimensional images suitable for speckle-tracking analysis. Longitudinal strain of the left ventricle was assessed from apical four-, two-, and three-chamber views, while circumferential strain was derived from basal, mid-ventricular, and apical short-axis views. Image analysis was performed using automated function imaging with the Q-Analysis module of the Philips QLAB 3.1 software.

According to the QLAB protocol, the left ventricular myocardium was automatically divided into seven segments for each apical projection. Peak systolic strain was defined as the maximal myocardial shortening during systole for both longitudinal and circumferential components. Global longitudinal strain and global circumferential strain were calculated as the average of segmental peak strain values and displayed using a standardized bull’s-eye representation. Early diastolic peak strain rate was derived from the corresponding strain curves for both deformation components [18].

Right ventricular global longitudinal strain (RV-GLS) was obtained by averaging segmental strain values derived from the apical four-chamber view. Right ventricular free wall longitudinal strain (RV-FWLS) was calculated as the mean strain of the basal, mid, and apical segments of the right ventricular lateral wall, excluding septal segments, in accordance with current recommendations [39].

Left atrial strain analysis was performed using a biplane approach. In both apical four- and two-chamber views, the left atrial endocardial border was automatically segmented into seven regions. Left atrial conduit strain (LAScd), contractile strain (LASct), and reservoir strain (LASr) were measured, with LASr defined as the sum of LAScd and LASct. Final LAS values were obtained by averaging measurements from the two apical views [40]. Strain rate analysis was additionally performed to quantify atrial deformation during ventricular systole, early diastole, and late diastole. Left atrial stiffness was estimated as the ratio between LASr and E/e′ [41].

Right atrial reservoir strain (RASr) was assessed by positioning reference points at the septal and lateral portions of the tricuspid annulus and along the superior right atrial endocardial border, following standardized methodology [42].

Finally, the time required to complete each speckle-tracking echocardiographic examination was recorded and expressed in minutes.

Normal reference values were defined as absolute strain values >20% for LV-GLS [33], >23.6% for LV-GCS [43], >20% for RV-GLS [44], >39% for LASr [45], and >44% for RASr [46].

### 2.5. Outcome Assessment

Patients were prospectively followed according to a predefined schedule including three study visits: at baseline, prior to ICI initiation, and at 1 month and 3 months after treatment start, with the primary aim of evaluating short-term changes in biventricular and biatrial myocardial mechanics. Longitudinal variations in myocardial deformation parameters were considered the main outcome of the study.

As additional outcome assessments, the study also explored factors associated with ICI–related left ventricular dysfunction, defined as a relative reduction in LV-GLS greater than 15% compared with baseline values [47]. Independent predictors of this strain-based ventricular dysfunction were evaluated.

Furthermore, the occurrence of adverse clinical events, including unplanned emergency department visits, episodes of clinical deterioration, and all-cause mortality, was assessed as an exploratory outcome. Independent predictors of these adverse events were also analyzed. In addition to scheduled visits, all unscheduled hospital admissions, clinical worsening, and deaths were systematically recorded from baseline evaluation through a mean observational period of 5.4 ± 3 months.

### 2.6. Statistical Analysis

Continuous variables were tested for normality using the Kolmogorov–Smirnov test and are presented as mean ± standard deviation or median with interquartile range, as appropriate. For clarity, myocardial strain values (LV-GLS, LV-GCS, and RV-GLS) are reported as absolute values.

Longitudinal changes in haemodynamic, laboratory, conventional echocardiographic, and myocardial strain parameters across baseline, 1-month, and 3-month follow-up were evaluated using repeated-measures ANOVA for normally distributed variables. Categorical variables assessed at multiple time points were compared using Cochran’s Q test.

Univariate logistic regression analysis was used to identify variables associated with ICI-related left ventricular dysfunction, defined as a relative reduction in LV-GLS >15% from baseline at 3 months. Cox proportional hazards regression analysis was performed to evaluate predictors of adverse clinical events and all-cause mortality during follow-up. Variables included in multivariable models were selected based on clinical relevance and prior evidence, while ensuring a parsimonious approach. Receiver operating characteristic (ROC) curve analysis was performed for variables significantly associated with study outcomes to identify optimal cut-off values using the Youden index.

Reproducibility of LV-GLS measurements was assessed using the intraclass correlation coefficient (ICC).

All statistical analyses were conducted using SPSS software (version 28; SPSS Inc., Chicago, IL, USA). A two-tailed *p* value < 0.05 was considered statistically significant.

## 3. Results

### 3.1. Baseline Demographic and Clinical Data

The baseline characteristics of the study population are reported in Table 1.

The cohort consisted of consecutive adult patients undergoing ICI therapy, with a moderate burden of cardiovascular risk factors. Hypertension and smoking were the most prevalent comorbidities, whereas diabetes mellitus, dyslipidemia, and established coronary artery disease were relatively uncommon.

Patients presented with a heterogeneous spectrum of advanced malignancies, most commonly breast cancer and melanoma. Approximately half of the cohort had metastatic disease at baseline, while the remaining patients received ICIs in the neoadjuvant or adjuvant setting. Most patients were treated with PD-1 inhibitor–based regimens, either as monotherapy or in combination, and a minority received concomitant anthracycline-based therapy.

Baseline cardiovascular medication use reflected the underlying clinical profile, with renin–angiotensin system inhibitors and beta-blockers being the most frequently prescribed agents.

### 3.2. Serial Hemodynamic and Laboratory Assessments

Table 2 summarizes hemodynamic parameters and laboratory findings at baseline (T0), 1-month (T1), and 3-month (T2) follow-up.

Hemodynamic variables, renal function indices, and biomarkers of myocardial injury remained overall stable throughout the study period.

In contrast, inflammatory markers showed a significant progressive increase over time, with RDW, NLR, and CRP reaching their highest values at 3 months. No significant longitudinal changes were observed in hemoglobin levels or natriuretic peptide concentrations.

### 3.3. Serial Conventional Echocardiographic Assessment

Conventional echocardiographic parameters at baseline and during follow-up are listed in Table 3.

Overall, conventional echocardiographic indices remained stable throughout the study period, with no significant longitudinal changes in left ventricular geometry, chamber dimensions, systolic or diastolic function, or right ventricular size and performance.

The prevalence of more than mild valvular regurgitation was low and did not significantly vary over time. Estimated sPAP also remained unchanged during follow-up.

### 3.4. Longitudinal Changes in Myocardial Strain Parameters

Table 4 details myocardial strain and strain rate parameters measured at baseline and during follow-up.

A significant and progressive impairment of left ventricular longitudinal mechanics was observed, with a consistent reduction in LV-GLS and its corresponding strain rate over time, whereas left ventricular circumferential strain parameters remained largely unchanged.

In parallel, a marked and early deterioration of biatrial mechanics was detected. Left and right atrial conduit, contractile, and reservoir strain values, as well as the corresponding strain rate indices, declined significantly during follow-up, accompanied by an increase in left atrial stiffness.

Right ventricular longitudinal function also progressively worsened, as reflected by reductions in RV-GLS and RV-FWLS, despite preserved conventional right ventricular systolic indices.

The time required to complete speckle-tracking analysis remained stable, supporting the feasibility of comprehensive biventricular and biatrial strain assessment in clinical practice.

**Figure 1:**
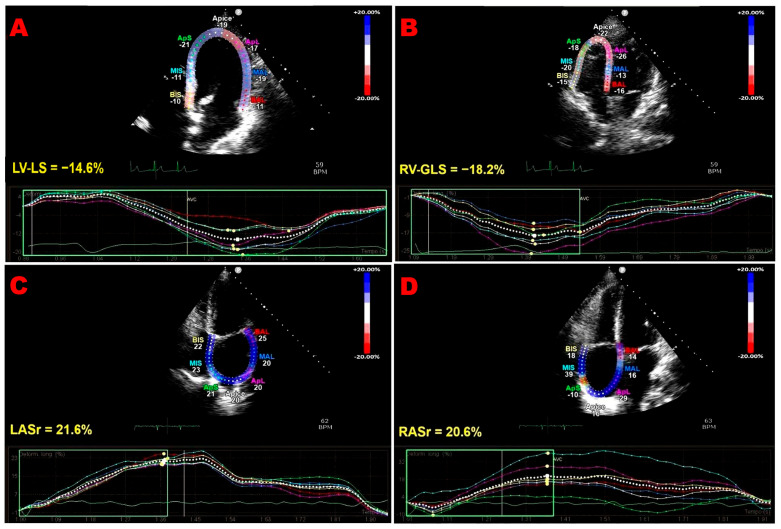
Representative examples of biventricular and biatrial myocardial strain parameters assessed by speckle-tracking echocardiography from the apical four-chamber (4C) view in an ICI-treated patient enrolled in the present study who developed ICI-related cardiac dysfunction. (**A**) LV-LS, left ventricular longitudinal strain. (**B**) RV-GLS, right ventricular global longitudinal strain. (**C**) LASr, left atrial reservoir strain. (**D**) RASr, right atrial reservoir strain. For each cardiac chamber, color-coded segmental strain maps are provided together with the corresponding strain curves. ICI, immune checkpoint inhibitors.

Figure 1 shows representative examples of biventricular and biatrial dysfunction identified by speckle-tracking echocardiography in an ICI-treated patient enrolled in the present study.

**Figure 2:**
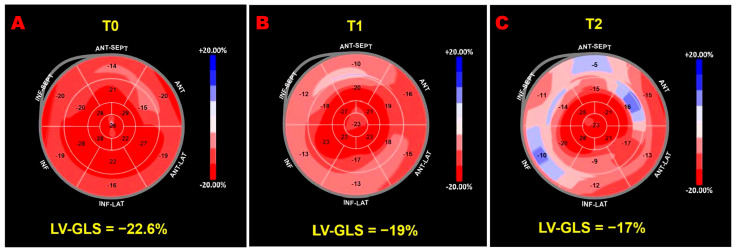
Speckle-tracking echocardiography–derived LV-GLS bull’s-eye plots obtained in a patient enrolled in the present study who developed ICI-related cardiotoxicity. (**A**) Normal LV-GLS bull’s-eye plot at baseline (T0). (**B**) Mild impairment of the LV-GLS bull’s-eye plot at the 1-month follow-up (T1). (**C**) Moderate impairment of the LV-GLS bull’s-eye plot at the 3-month follow-up (T2). ICI, immune checkpoint inhibitors; LV-GLS, left ventricular global longitudinal strain.

Figure 2 illustrates serial LV-GLS bull’s-eye plots from an ICI-treated patient who developed a >15% reduction in LV-GLS compared with baseline.

### 3.5. Predictors of Subclinical Left Ventricular Dysfunction

At the 3-month follow-up (T2), subclinical left ventricular dysfunction—defined as a relative reduction in LV-GLS greater than 15% compared with baseline [47]—was observed in 24 out of 28 patients (85.7%). The results of logistic regression analyses evaluating predictors of ICI-related left ventricular dysfunction are presented in Table 5 and Table 6.

Table 5 (Model 1) reports the logistic regression analysis including baseline demographic and anthropometric variables. In this model, age was significantly associated with LV-GLS impairment, whereas sex and baseline BMI were not independently associated with the outcome.

ROC curve analysis indicated that an age ≥46 years was associated with LV-GLS impairment in ICI-treated patients, with 96% sensitivity and 100% specificity (AUC = 0.99; 95% CI, 0.96–1.00; *p* = 0.002).

Table 6 (Model 2) presents the logistic regression analysis including variables assessed at 3-month follow-up. In this model, NLR measured at T2 emerged as a significant predictor of LV-GLS impairment, while systolic blood pressure and exposure to pembrolizumab therapy were not independently associated with treatment-related left ventricular dysfunction.

ROC curve analysis demonstrated that a T2-NLR (measured at the 3-month follow-up) ≥ 4 predicted LV-GLS impairment in ICI-treated patients with 100% sensitivity and 100% specificity (AUC = 1.00; 95% CI, 1.00–1.00; *p* = 0.002).

Overall, these findings suggest that both older age and increased systemic inflammatory burden during follow-up are associated with a higher likelihood of developing subclinical ICI-related left ventricular dysfunction.

### 3.6. Cox Regression Analyses for Adverse Clinical Events and Mortality

During a mean follow-up period of 5.4 ± 3 months, a total of 15 adverse clinical events were recorded. Five patients died during follow-up due to progression of the underlying malignancy, with no deaths attributable to immune-related cardiovascular adverse events. No fatal cardiac events or cases of fulminant myocarditis were recorded. Among non-fatal events, three patients required emergency department admission for worsening dyspnea associated with pleural effusion, three for sepsis related to urinary or respiratory tract infections, two for severe anemia, and two for atrial fibrillation complicated by hemodynamic decompensation.

The results of univariate and multivariate Cox proportional hazards regression analyses evaluating predictors of adverse clinical events and/or all-cause mortality during follow-up are presented in Table 7, Table 8 and Table 9.

Table 7 (Model 1) reports the Cox regression analysis including baseline demographic variables and baseline left ventricular myocardial deformation. In this model, both age and baseline left ventricular global longitudinal strain (T0-LV-GLS) were independently associated with the risk of adverse clinical outcomes. Increasing age was associated with a higher hazard of events, whereas more preserved baseline LV-GLS values were associated with a significantly lower risk, highlighting the prognostic relevance of baseline myocardial mechanics.

ROC curve analysis demonstrated that an age ≥ 67 years predicted short-term adverse clinical outcomes in ICI-treated patients with 93% sensitivity and 85% specificity (AUC = 0.95; 95% CI, 0.87–1.00; *p* < 0.001). In comparison, a baseline LV-GLS ≤ 19.5% predicted short-term adverse clinical outcomes with 100% sensitivity and 100% specificity (AUC = 1.00; 95% CI, 1.00–1.00; *p* < 0.001).

Table 8 (Model 2) presents the Cox regression analysis evaluating baseline systemic inflammatory status. In this model, age remained independently associated with adverse clinical outcomes, while baseline neutrophil-to-lymphocyte ratio (T0-NLR), although significant at univariate analysis, did not retain independent prognostic significance after multivariable adjustment.

A third Cox regression model (Model 3) was constructed to explore the prognostic impact of cancer stage. In this univariate model, age remained significantly associated with adverse clinical outcomes, whereas the presence of metastatic cancer at baseline was not significantly associated with the risk of adverse events or mortality (Table 9).

Overall, these findings indicate that baseline left ventricular longitudinal mechanics provide incremental prognostic information for short-term adverse clinical outcomes in patients undergoing ICI therapy, whereas baseline inflammatory markers appear to have a weaker independent association.

### 3.7. Measurement Variability

Reproducibility of LV-GLS measurements was assessed by evaluating both intraobserver and interobserver variability in a predefined subgroup of 15 cancer patients. Overall, agreement was found to be excellent. Intraobserver reliability showed a very high level of concordance, as reflected by an ICC of 0.97 (95% confidence interval [CI], 0.94–0.99). Similarly, interobserver agreement was also strong, with an ICC of 0.91 (95% CI, 0.79–0.97). These findings support the robustness and reliability of LV-GLS assessment in the present study. Detailed results are provided in the Supplementary Material S1.

## 4. Discussion

### 4.1. Principal Findings

In this prospective, monocentric study of patients with advanced cancer undergoing ICI therapy, we observed a consistent pattern of early subclinical myocardial dysfunction affecting both ventricles and both atria, despite preserved conventional echocardiographic parameters. Over a three-month follow-up, the vast majority of patients developed a significant reduction in LV-GLS, reflecting a progressive impairment of longitudinal myocardial mechanics, while LVEF, chamber dimensions, and diastolic indices remained stable. These deformation abnormalities occurred in parallel with a gradual and significant increase in systemic inflammatory indices, including NLR and CRP, whereas circulating markers of myocardial injury and wall stress, such as high-sensitivity troponin T and NT-proBNP, did not show significant longitudinal changes. In contrast, left ventricular circumferential mechanics remained largely preserved, as neither global nor segmental circumferential strain or strain rate showed significant changes over time, suggesting a selective vulnerability of longitudinal fibers in the early phase of ICI-related myocardial involvement. In parallel, right ventricular longitudinal mechanics progressively deteriorated, indicating early biventricular involvement.

Beyond ventricular mechanics, a key novel finding of the present study is the consistent and marked impairment of biatrial myocardial function. Both left and right atrial reservoir, conduit, and contractile strain parameters declined significantly during follow-up, accompanied by worsening atrial strain rate indices and increased left atrial stiffness. These changes occurred in the absence of significant atrial enlargement, highlighting the sensitivity of STE in detecting early atrial dysfunction.

Importantly, exploratory regression analyses identified older age and greater systemic inflammatory burden as significant correlates of ICI-related left ventricular strain impairment, while baseline LV-GLS emerged as a robust independent predictor of adverse clinical events and mortality during follow-up. All deaths observed during the study period were attributable to progression of the underlying malignancy, with no fatal immune-related cardiovascular adverse events recorded. Notably, cancer stage at baseline, including the presence of metastatic disease, did not independently influence short-term adverse clinical outcomes, indicating that early prognosis in ICI-treated patients may be more closely driven by myocardial functional vulnerability than by oncologic disease burden.

In the present cohort, no cardioprotective therapy was initiated during follow-up, as patients remained clinically and hemodynamically stable and preserved LVEF throughout the observation period. Although subclinical myocardial dysfunction was frequently detected by STE, it did not translate into overt heart failure or clinically significant systolic impairment during short-term follow-up, suggesting that early ICI-related myocardial involvement may remain clinically manageable when promptly identified and closely monitored.

### 4.2. Comparison with Previous Studies

Our findings are largely consistent with previous echocardiographic studies investigating the impact of ICI therapy on myocardial deformation, while also extending current knowledge by providing a comprehensive evaluation of biventricular and biatrial mechanics.

Most available studies [24,25,26,27,28,29,30,31,32] have primarily focused on LV-GLS, which was assessed in the vast majority of published reports, whereas circumferential and radial strain parameters, as well as atrial and right ventricular strain, were evaluated far less frequently. Across these studies, a significant deterioration of LV-GLS following ICI therapy has been commonly reported, with relative reductions ranging approximately between 9% and 19%, despite minimal or absent changes in conventional indices such as LVEF [48].

In line with this literature, our study demonstrated a significant and progressive reduction in LV-GLS over short-term follow-up, while LVEF and other standard echocardiographic parameters remained preserved. Importantly, the absence of significant changes in LV-GCS in our cohort is consistent with observations from other clinical settings, in which GLS impairment typically precedes GCS deterioration [49,50]. This temporal dissociation reflects the pivotal role of long-axis function in left ventricular systolic performance, which contributes approximately 60% of total stroke volume [51]. Moreover, longitudinally oriented subendocardial fibers exhibit higher oxygen consumption and are particularly vulnerable to ischemia and inflammatory injury [52]. As a result, longitudinal strain is altered in the early phases of various cardiac diseases and is generally considered more sensitive than circumferential or radial strain for detecting subclinical myocardial dysfunction [53].

It is noteworthy that a small number of prior investigations did not observe significant LV-GLS changes after ICI exposure [26,32]; however, these discrepancies may reflect heterogeneity in study design, imaging timing, cancer type, or baseline cardiovascular risk profile. Importantly, our data are concordant with studies reporting that strain-based metrics detect myocardial involvement earlier and more sensitively than conventional echocardiography.

Previous echocardiographic studies that simultaneously assessed left and right ventricular mechanics have described a concomitant impairment of biventricular longitudinal function after ICI therapy, supporting the concept of diffuse myocardial involvement [24,26,29]. Our findings confirm and reinforce this observation, showing parallel deterioration of LV-GLS, RV-GLS, and RV-FWLS, even in the absence of overt right ventricular systolic dysfunction as assessed by TAPSE.

Data on atrial mechanics in ICI-treated patients remain particularly scarce. Only a limited number of studies have evaluated atrial strain [26], and even fewer have explored biatrial function. In this context, the present study provides novel evidence of early and marked impairment of both left and right atrial mechanics, involving reservoir, conduit, and contractile phases. These findings expand previous observations and suggest that atrial dysfunction may represent an integral component of ICI-related subclinical cardiotoxicity, rather than a secondary phenomenon related solely to ventricular impairment.

Unlike some prior reports, no cases of clinically overt myocarditis were documented in our cohort. This supports the concept that significant myocardial deformation abnormalities may occur independently of overt inflammatory cardiomyopathy, reinforcing the role of strain imaging as a sensitive tool for detecting early and potentially reversible myocardial injury in patients receiving ICIs.

### 4.3. Pathophysiological Mechanisms Underpinning Subclinical Myocardial Dysfunction in ICI-Treated Patients

Growing experimental evidence suggests that ICI therapy promotes a systemic and myocardial pro-inflammatory milieu that may lead to early subclinical myocardial dysfunction, even in the absence of overt myocarditis. Preclinical studies have shown that short-term ICI exposure induces T-cell hyperactivation, resulting in cardiomyocyte injury and the release of pro-inflammatory cytokines, including interleukins (IL-1α, IL-1β, IL-6, IL-17α), interferon-γ, and tumor necrosis factor-α [54]. These inflammatory pathways have been associated with myocardial strain impairment and activation of profibrotic processes, such as increased expression of galectin-3, procollagen type I, and matrix metalloproteinases, ultimately favoring early myocardial remodeling [54].

Consistent with these observations, cardiac magnetic resonance feature-tracking studies have demonstrated early and diffuse myocardial edema in ICI-treated patients, likely reflecting immune cell infiltration [4]. Persistent inflammation may subsequently promote interstitial fibrosis, leading to increased myocardial stiffness and reduced myocardial deformation, as reflected by lower strain values [23,55,56,57].

Experimental models also indicate heterogeneous cardiovascular effects among different immune checkpoint targets. Anti–PD-1 therapy has been associated with greater NF-κB activation, cardiomyocyte hypertrophy, and vascular inflammation compared with CTLA-4 inhibition [54]. In addition, ICI-related cardiotoxicity may be indirectly mediated by gut microbiota alterations, including dysbiosis, reduced short-chain fatty acid production, and macrophage polarization toward a pro-inflammatory phenotype, thereby amplifying myocardial inflammation and cardiomyocyte apoptosis [58].

Beyond direct myocardial injury, ICIs exacerbate vascular and endothelial inflammation, accelerating atherosclerotic plaque progression and endothelial activation in preclinical models [59,60]. Although acute ICI-related myocarditis represents the most severe immune-mediated cardiac manifestation, it remains rare. Nevertheless, mechanistic overlap likely exists between fulminant myocarditis and subclinical myocardial involvement, both characterized by immune tolerance breakdown, T-cell expansion, macrophage infiltration, and cytokine-mediated myocardial injury [61,62,63].

As myocardial stiffness increases, myocardial deformation progressively declines across multiple cardiac chambers. This diffuse pattern of strain impairment, involving both ventricles and atria, has been reported in other clinical settings [64,65,66,67] and is consistent with our findings. Right ventricular involvement may occur early, potentially due to its smaller myocardial mass and thinner wall structure [26,68], while ventricular interdependence further contributes to RV strain alterations [69]. Finally, although data on ICI-related biatrial dysfunction remain limited, early impairment of atrial reservoir function may reflect increased myocardial stiffness and altered ventricular–atrial coupling, as described in conditions associated with chronic pressure overload [70,71].

Overall, these data support inflammation-driven myocardial injury as a central mechanism underlying subclinical biventricular and biatrial dysfunction in ICI-treated patients and provide a biological rationale for the use of advanced myocardial deformation imaging in early cardio-oncology surveillance.

### 4.4. Clinical Translation of the Findings

Taken together, the results of the present study further reinforce the clinical value of myocardial strain imaging for the early detection of subclinical cardiotoxicity in patients treated with ICIs. In our cohort, a marked reduction in LV-GLS was observed in the majority of patients within a short time frame, despite preserved LVEF and stable conventional echocardiographic parameters. This finding is consistent with the well-established role of LV-GLS as a sensitive and highly reproducible marker of left ventricular systolic function [72,73,74], capable of identifying early myocardial impairment even in the presence of preserved LVEF [75,76]. Importantly, in our study baseline LV-GLS also emerged as an independent predictor of adverse clinical events and mortality, underscoring its prognostic relevance beyond diagnostic purposes [77,78,79].

Current ESC cardio-oncology guidelines recommend baseline LV-GLS assessment in patients receiving potentially cardiotoxic therapies and identify a relative GLS reduction of approximately 15% as a threshold suggestive of subclinical myocardial dysfunction during cancer treatment [47]. Our findings strongly support the applicability of this cut-off in the setting of ICI therapy, where a high prevalence of GLS decline was observed early after treatment initiation. Moreover, the concomitant deterioration of right ventricular longitudinal strain and the consistent impairment of biatrial mechanics observed in our cohort indicate that ICI-related cardiotoxicity is not confined to the left ventricle, but rather reflects a more diffuse myocardial process.

In this context, reliance on LVEF alone appears insufficient for early cardiovascular surveillance in ICI-treated patients. Comprehensive strain imaging, including biventricular and biatrial assessment, may allow a more accurate characterization of myocardial involvement and facilitate earlier identification of patients at higher risk. The association observed between systemic inflammatory burden and LV-GLS impairment further suggests that integrating imaging findings with inflammatory biomarkers could enhance risk stratification. Early detection of strain abnormalities, even in asymptomatic patients, may therefore prompt closer monitoring and consideration of timely therapeutic interventions—such as optimization of cardioprotective therapy, corticosteroid initiation, or modification of oncologic treatment—potentially reducing the risk of progression toward overt myocarditis or other severe cardiovascular immune-related adverse events. Nonetheless, prospective studies are still needed to define optimal surveillance intervals and management strategies in this emerging clinical scenario.

### 4.5. Methodological Strengths and Limitations

The present study has several notable methodological strengths. It was conducted in a prospective manner with consecutive patient inclusion, reflecting a real-world cardio-oncology population. All transthoracic echocardiographic examinations were performed using a standardized protocol by a single experienced operator, minimizing inter-observer variability. The study provides a comprehensive and integrated assessment of myocardial mechanics, encompassing left and right ventricular as well as biatrial strain and strain-rate parameters, which remain poorly characterized in patients treated with ICIs. Moreover, serial evaluations at predefined time points enabled the detection of early and progressive subclinical myocardial dysfunction despite preserved conventional echocardiographic indices. Finally, the integration of myocardial deformation parameters with short-term clinical outcomes offers clinically relevant insights for cardiovascular risk stratification in ICI-treated patients.

Several limitations should be acknowledged. The relatively small sample size and monocentric design may limit the generalizability of the findings, and the short-term follow-up precludes assessment of the long-term evolution of myocardial deformation abnormalities. Owing to the observational nature of the study, causal relationships between ICI therapy and myocardial strain impairment cannot be definitively established, and potential confounding effects related to the underlying malignancy and its inflammatory burden cannot be excluded. In addition, oncologic response to therapy was not systematically assessed.

Finally, several limitations intrinsic to STE should be considered. Myocardial strain measurements are influenced by image quality and frame rate [80], operator expertise and tracking accuracy [81], inter-vendor and software-related variability [82], and load-dependent changes in blood pressure and intravascular volume [83]. Patient-related factors, including acoustic window quality and body habitus, may further affect tracking feasibility, particularly in patients with advanced malignancy [84].

## 5. Conclusions

In this prospective monocentric cohort, short-term ICI therapy was associated with early subclinical myocardial dysfunction involving both ventricles and atria, despite preserved conventional echocardiographic parameters. Over three months, LV-GLS declined in most patients, accompanied by impairment of right ventricular longitudinal mechanics and biatrial strain, with increased left atrial stiffness. Older age and higher inflammatory burden were associated with LV-GLS deterioration, while baseline LV-GLS provided prognostic information for adverse clinical events. These findings support the use of comprehensive biventricular and biatrial strain assessment for early cardio-oncology surveillance in ICI-treated patients and warrant larger studies to define long-term clinical implications.

## Figures and Tables

**Table 1:** Baseline clinical characteristics of ICI-treated patients included in the present study. Continuous variables are expressed as mean ± standard deviation, whereas categorical variables are expressed as number (percentage). ACEi-ARBs, angiotensin-converting enzyme inhibitors/angiotensin receptor blockers; BMI, body mass index; BSA, body surface area; CAD, coronary artery disease; CCB, calcium channel blockers; ICI, immune checkpoint inhibitors.

	ICI-Treated Patients (n = 28)
**Demographics and anthropometrics**	
Age (yrs)	62.1 ± 16.0
Male sex (%)	12 (42.8)
BSA (m^2^)	1.73 ± 0.19
BMI (kg/m^2^)	23.0 ± 3.5
**Cardiovascular risk factors and cardiovascular comorbidities**	
Smoking (%)	9 (32.1)
Hypertension (%)	13 (46.4)
Type 2 diabetes (%)	3 (10.7)
Dyslipidemia (%)	2 (7.1)
History of CAD (%)	2 (7.1)
**Cancer type**	
Melanoma (%)	6 (21.4)
Breast (%)	10 (35.7)
Lung (%)	4 (14.3)
Colorectal (%)	2 (7.1)
Kidney (%)	3 (10.7)
Esophagus (%)	3 (10.7)
Metastatic cancer (%)	14 (50.0)
**ICI therapy**	
Pembrolizumab (%)	18 (64.3)
Nivolumab (%)	9 (32.1)
Pembrolizumab plus Relatlimab (%)	3 (10.7)
Durvalumab (%)	1 (3.6)
Co-treatment with antracyclines (%)	7 (25.0)
Neoadjuvant ICI (%)	7 (25.0)
Adjuvant ICI (%)	7 (25.0)
ICI for metastatic cancer (%)	14 (50.0)
**Current cardiac treatment**	
Anticoagulants (%)	1 (3.6)
Antiplatelets (%)	7 (25.0)
ACEi-ARBs (%)	12 (42.8)
Beta blockers (%)	10 (35.7)
Antiarrhythmics (%)	1 (3.6)
CCB (%)	7 (25.0)
Diuretics (%)	7 (25.0)
Statins (%)	1 (3.6)

**Table 2:** Hemodynamic parameters and laboratory findings at baseline and during follow-up in ICI-treated patients. All variables are expressed as continuous values and were compared across time points using repeated-measures ANOVA; statistically significant *p* values are shown in bold. DBP, diastolic blood pressure; eGFR, estimated glomerular filtration rate; Hb, hemoglobin; Hs-TnT, high-sensitivity troponin T; ICI, immune checkpoint inhibitors; NLR, neutrophil-to-lymphocyte ratio; NT pro-BNP, N-terminal pro–B-type natriuretic peptide; PCR, C-reactive protein; RDW, red cell distribution width; SBP, systolic blood pressure.

	T0	T1	T2	*p*-Value
**Hemodynamics**				
Heart rate (bpm)	75.1 ± 11.6	75.9 ± 11.6	76.4 ± 14.7	0.96
SBP (mmHg)	132.4 ± 24.5	126.1 ± 20.1	125.4 ± 25.1	0.74
DBP (mmHg)	72.7 ± 13.1	69.5 ± 6.3	68.5 ± 10.9	0.61
**Blood tests**				
Hb (g/dL)	12.7 ± 2.0	12.3 ± 1.7	12.0 ± 1.8	0.76
RDW (%)	13.8 ± 1.8	15.2 ± 2.6	16.8 ± 2.9	**0.002**
NLR	1.8 ± 0.9	2.9 ± 1.5	4.2 ± 2.4	**<0.001**
Creatinine (mg/dL)	0.75 ± 0.21	0.74 ± 0.22	0.81 ± 0.25	0.65
eGFR (mL/min/m^2^)	104.5 ± 13.6	103.4 ± 10.5	98.4 ± 16.6	0.55
PCR (mg/dL)	4.4 ± 3.5	13.9 ± 20.0	33.4 ± 44.3	**0.03**
Hs-TnT (ng/L)	4.2 ± 3.3	5.0 ± 3.9	5.5 ± 4.0	0.68
NT pro-BNP (pg/mL)	300.6 ± 319.7	575.8 ± 1305.8	416.3 ± 436.0	0.69

**Table 3:** Conventional echoDoppler parameters at baseline and during follow-up in ICI-treated patients. Continuous variables are expressed as mean ± standard deviation and were compared across time points using repeated-measures ANOVA; categorical variables are expressed as number (percentage). AR, aortic regurgitation; E/A, early-to-late diastolic transmitral flow velocity ratio; e′, early diastolic mitral annular velocity; ICI, immune checkpoint inhibitors; IVS, interventricular septal thickness; LAVi, left atrial volume index; LV, left ventricular; LVEDD, left ventricular end-diastolic diameter; LVEDV, left ventricular end-diastolic volume; LVEF, left ventricular ejection fraction; LVESV, left ventricular end-systolic volume; LVMi, left ventricular mass index; MR, mitral regurgitation; PW, posterior wall thickness; RVIT, right ventricular inflow tract diameter; RWT, relative wall thickness; sPAP, systolic pulmonary artery pressure; TAPSE, tricuspid annular plane systolic excursion; TR, tricuspid regurgitation.

Conventional EchoDoppler Indices	T0	T1	T2	*p*-Value
IVS (mm)	11.1 ± 3.0	11.3 ± 3.3	11.3 ± 3.2	0.97
PW (mm)	8.1 ± 1.5	8.1 ± 1.4	8.3 ± 1.6	0.90
LVEDD (mm)	45.3 ± 4.8	45.2 ± 4.4	45.3 ± 4.9	0.99
RWT	0.37 ± 0.07	0.36 ± 0.07	0.37 ± 0.08	0.96
LVMi (g/m^2^)	87.3 ± 26.5	87.9 ± 31.4	91.0 ± 39.4	0.93
Normal LV geometric model (%)	21 (75.0)	21 (75.0)	19 (67.8)	0.13
LV Concentric remodeling (%)	1 (3.6)	1 (3.6)	3 (3.6)	0.13
LV concentric hypertrophy (%)	3 (10.7)	3 (10.7)	1 (3.6)	0.13
LV eccentric hypertrophy (%)	3 (10.7)	3 (10.7)	5 (17.8)	0.13
LVESV (mL)	67.0 ± 16.7	63.6 ± 13.3	67.8 ± 16.1	0.67
LVEDV (mL)	23.7 ± 6.6	23.7 ± 5.4	25.5 ± 8.5	0.65
LVEF (%)	64.5 ± 4.7	62.5 ± 4.1	62.6 ± 6.6	0.42
E/A ratio	1.0 ± 0.3	0.8 ± 0.2	0.9 ± 0.3	0.23
E/average e’ ratio	8.5 ± 4.5	9.2 ± 3.9	10.5 ± 5.0	0.38
LAVi (mL/m^2^)	32.0 ± 9.5	31.2 ± 8.1	34.0 ± 9.2	0.60
More than mild MR (%)	6 (21.4)	6 (21.4)	8 (28.6)	0.13
More than mild AR (%)	0 (0.0)	0 (0.0)	1 (3.6)	0.37
More than mild TR (%)	0 (0.0)	1 (3.6)	1 (3.6)	0.37
RVIT (mm)	29.0 ± 3.4	28.7 ± 2.9	29.7 ± 2.6	0.57
TAPSE (mm)	24.0 ± 3.5	22.3 ± 3.5	21.8 ± 4.4	0.17
sPAP (mmHg)	26.9 ± 3.4	27.6 ± 3.7	28.9 ± 3.3	0.22

**Table 4:** Longitudinal changes in myocardial strain and strain rate parameters at baseline and during follow-up in ICI-treated patients. Continuous variables are expressed as mean ± standard deviation and evaluated longitudinally using repeated-measures ANOVA; statistically significant *p* values are denoted in bold. 2C, two-chamber view; 3C, three-chamber view; 4C, four-chamber view; CS, circumferential strain; CSR, circumferential strain rate; FWLS, free wall longitudinal strain; GLS, global longitudinal strain; GLSR, global longitudinal strain rate; GCS, global circumferential strain; GCSR, global circumferential strain rate; ICI, immune checkpoint inhibitors; LA, left atrial; LAcd, left atrial conduit strain; LAct, left atrial contractile strain; LASr, left atrial reservoir strain; LS, longitudinal strain; LSR, longitudinal strain rate; LV, left ventricular; RA, right atrial; RAcd, right atrial conduit strain; RAct, right atrial contractile strain; RASr, right atrial reservoir strain; RV, right ventricular; STE, speckle-tracking echocardiography.

Myocardial Strain Parameters	T0	T1	T2	*p*-Value
LS-4C (%)	20.9 ± 2.3	19.4 ± 3.4	17.9 ± 3.1	**0.01**
LSR-4C (s^−1^)	1.05 ± 0.17	1.04 ± 0.21	0.89 ± 0.23	**0.02**
LS-2C (%)	21.2 ± 2.7	19.1 ± 3.4	17.9 ± 3.0	**0.01**
LSR-2C (s^−1^)	1.08 ± 0.17	1.05 ± 0.18	0.87 ± 0.21	**0.004**
LS-3C (%)	20.1 ± 3.7	17.4 ± 3.1	16.5 ± 2.8	**0.005**
LSR-3C (s^−1^)	1.13 ± 0.17	1.04 ± 0.14	0.95 ± 0.14	**0.004**
LV-GLS (%)	20.7 ± 2.1	18.9 ± 3.0	17.6 ± 2.7	**0.002**
LV-GLSR (s^−1^)	1.08 ± 0.14	1.06 ± 0.15	0.88 ± 0.20	**0.001**
Basal LV-CS (%)	18.7 ± 3.5	17.6 ± 5.0	17.0 ± 4.6	0.56
Basal LV-CSR (s^−1^)	1.24 ± 0.15	1.18 ± 0.27	1.11 ± 0.22	0.23
Mid LV-CS (%)	22.7 ± 3.6	20.3 ± 5.1	20.7 ± 4.9	0.31
Mid LV-CSR (s^−1^)	1.39 ± 0.17	1.35 ± 0.20	1.32 ± 0.22	0.64
Apical LV-CS (%)	27.8 ± 5.4	25.7 ± 5.6	25.2 ± 7.8	0.47
Apical LV-CSR (s^−1^)	1.82 ± 0.31	1.80 ± 0.47	1.62 ± 0.49	0.31
LV-GCS (%)	22.8 ± 3.3	20.7 ± 4.5	20.5 ± 5.3	0.29
LV-GCSR (s^−1^)	1.46 ± 0.14	1.45 ± 0.27	1.32 ± 0.21	0.13
LAcd strain (%)	26.1 ± 5.2	24.0 ± 5.8	20.1 ± 4.2	**0.005**
LAct strain (%)	9.5 ± 5.5	7.3 ± 4.7	4.2 ±3.0	**0.006**
LASr (%)	35.7 ±6.5	30.8 ±6.0	26.7 ± 5.5	**<0.001**
LASr/E/e’ ratio	5.1 ± 2.7	3.7 ± 1.6	3.1 ± 1.8	**0.02**
LA-SRs (s^−1^)	1.72 ± 0.35	1.54 ± 0.37	1.22 ± 0.32	**<0.001**
LA-SRe (s^−1^)	1.94 ± 0.66	1.66 ± 0.77	1.26 ± 0.61	**0.02**
LA-SRl (s^−1^)	2.73 ± 0.62	2.51 ± 0.49	1.87 ± 0.41	**<0.001**
RAcd strain (%)	31.6 ± 7.7	26.7 ± 4.9	21.6 ± 6.2	**<0.001**
RAct strain (%)	10.2 ± 5.8	8.3 ± 5.8	4.3 ± 2.9	**0.009**
RASr (%)	41.5 ± 9.0	34.3 ± 4.9	26.4 ± 4.3	**<0.001**
RA-SRs (s^−1^)	2.01 ± 0.54	1.88 ± 0.36	1.63 ± 0.35	**0.04**
RA-SRe (s^−1^)	1.76 ± 0.34	1.60 ± 0.26	1.40 ± 0.43	**0.04**
RA-SRl (s^−1^)	2.19 ± 0.66	2.08 ± 0.47	1.68 ± 0.40	**0.04**
RV-GLS (%)	20.1 ± 3.7	19.2 ± 3.3	16.6 ± 4.1	**0.03**
RV-GLSR (s^−1^)	1.27 ± 0.26	1.08 ± 0.14	1.01 ± 0.21	**0.005**
RV-FWLS (%)	21.2 ± 3.7	19.2 ± 3.3	17.6 ± 4.3	**0.03**
Timing STE (min)	8.9 ± 1.4	8.4 ± 1.4	8.1 ± 1.5	0.29

**Table 5:** Logistic regression analysis (Model 1) evaluating baseline demographic and anthropometric predictors of LV-GLS impairment. Statistically significant *p* values are highlighted in bold. OR, odds ratio; CI, confidence interval; BMI, body mass index; LV-GLS, left ventricular global longitudinal strain.

Model 1	Logistic Regression Analysis	
	OR (95% CI)	*p*-Value
**Age (yrs)**	1.72 (1.00–2.93)	**0.04**
**Male sex**	2.54 (0.23–28.0)	0.45
**Basal BMI (Kg/m^2^)**	1.13 (0.83–1.56)	0.43

**Table 6:** Logistic regression analysis (Model 2) evaluating 3-month follow-up variables associated with LV-GLS impairment. Statistically significant *p* values are reported in bold type. OR, odds ratio; CI, confidence interval; LV-GLS, left ventricular global longitudinal strain; NLR, neutrophil-to-lymphocyte ratio; SBP, systolic blood pressure; T2, 3-month follow-up.

Model 2	Logistic Regression Analysis	
	OR (95% CI)	*p*-Value
**T2-SBP (mmHg)**	1.05 (0.98–1.12)	0.17
**T2-NLR**	1.94 (1.25–3.01)	**0.003**
**Pembrolizumab therapy**	3.00 (0.34–26.2)	0.32

**Table 7:** Cox proportional hazards regression analysis (Model 1) evaluating baseline demographic variables and baseline left ventricular myocardial deformation as predictors of adverse clinical outcomes. Significant *p* values are shown in bold. HR, hazard ratio; CI, confidence interval; LV-GLS, left ventricular global longitudinal strain; T0, baseline.

Model 1	Univariate Cox Regression Analysis		Multivariate Cox Regression Analysis	
	HR (95% CI)	*p*-Value	HR (95% CI)	*p*-Value
**Age (yrs)**	1.11 (1.04–1.18)	**<0.001**	1.08 (1.01–1.16)	**0.03**
**T0-LV-GLS**	0.41 (0.27–0.62)	**<0.001**	0.43 (0.26–0.71)	**<0.001**

**Table 8:** Cox proportional hazards regression analysis (Model 2) evaluating baseline systemic inflammatory status as a predictor of adverse clinical outcomes. *p* values in bold denote statistical significance. HR, hazard ratio; CI, confidence interval; NLR, neutrophil-to-lymphocyte ratio; T0, baseline.

Model 2	Univariate Cox Regression Analysis		Multivariate Cox Regression Analysis	
	HR (95% CI)	*p*-Value	HR (95% CI)	*p*-Value
**Age (yrs)**	1.11 (1.04–1.18)	**<0.001**	1.09 (1.01–1.17)	**0.02**
**T0-NLR**	1.54 (1.21–1.96)	**<0.001**	1.15 (0.84–1.58)	0.37

**Table 9:** Univariate Cox proportional hazards regression analysis (Model 3) evaluating age and metastatic cancer status as predictors of adverse clinical outcomes in patients undergoing immune checkpoint inhibitor therapy. Hazard ratios (HRs) are reported with corresponding 95% confidence intervals (CIs). Statistically significant *p* values are shown in bold.

Model 3	Univariate Cox Regression Analysis	
	HR (95% CI)	*p*-Value
**Age (yrs)**	1.11 (1.04–1.18)	**<0.001**
**Metastatic cancer**	1.31 (0.47–3.60)	0.61

## Data Availability

Data extracted from included studies will be publicly available on Zenodo (https://zenodo.org).

## Supplementary Materials

The following supporting information can be downloaded at: https://www.mdpi.com/article/10.3390/jcm15020762/s1, Supplementary Material S1: LV-GLS measurement variability in cancer patients.
