# Supplementary material for: Effect of Immune Checkpoint Inhibitor Therapy on Biventricular and Biatrial Mechanics in Patients with Advanced Cancer: A Short-Term Follow-Up Study
Source: J Clin Med. 2026 Jan 16;15(2):762. doi: 10.3390/jcm15020762 (PMC12841717; doi:10.3390/jcm15020762)
Supplement: Supplementary file 1 [file jcm-15-00762-s001.zip › jcm-4096208-supplementary.pdf]

LV-GLS assessment			
PATIENT LIST	INITIAL MEASUREMENT	REMEASUREMENTS	
		Rater 1	Rater 2
1) P.M.	19.5	19.6	19.8
2) S.H.	18.7	19	19.2
3) S.B.	17.3	17.5	18
4) F.Z.	21	21.2	21.4
5) L.S.	20.9	20	19.5
6) M.G.	21	21.5	22
7) F.C.	20.2	21	22
8) R.S.	18.8	19.5	20
9) S.R.	24.5	26	28
10) G.S.	22.1	22.5	23
11) M.P.	19.4	19.6	19.8
12) M.L.	20.3	20.5	21
13) M.B.	21.4	21.8	22
14) C.C.	20.3	20.8	21
15) A.F.	22.6	23	23.5
ICC (95%CI)		0.97 (0.94-0.99)	0.91 (0.79-0.97)
